# Sex and Gender in Chronic Obstructive Pulmonary Disease: Does It Matter?

**DOI:** 10.3390/jpm16030152

**Published:** 2026-03-06

**Authors:** Christos Kyriakopoulos, Georgios Hillas, Antonia Assioura, Anastasia Papanikolaou, Vasileios Angelopoulos, Konstantinos Kostikas, Athena Gogali

**Affiliations:** 1 Respiratory Medicine Department, Faculty of Medicine, University of Ioannina, 45500 Ioannina, Greece; c.kyriako@uoi.gr (C.K.); antonia.assioura@gmail.com (A.A.); anastasiapapanikolaou13@gmail.com (A.P.); vasilios.angelop@gmail.com (V.A.); ktkostikas@uoi.gr (K.K.); 2 2nd Respiratory Medicine Department, “Attikon” University Hospital, National and Kapodistrian University of Athens, 12462 Athens, Greece; ghillas@med.uoa.gr

**Keywords:** COPD, sex, gender, personalized COPD management

## Abstract

Chronic obstructive pulmonary disease (COPD) is a major contributor to global respiratory morbidity and exhibits substantial sex- and gender-related differences in incidence, phenotype, pathophysiology, and outcomes across the life course. Historically regarded as a predominantly male disease due to higher smoking rates, COPD is now increasingly recognized among women, reflecting changing exposure patterns and enhanced diagnostic attention. Moreover, evidence indicates that women may be more biologically susceptible to the harmful effects of tobacco smoke and often develop COPD at younger ages. Clinical manifestations also differ, with women more frequently reporting dyspnea, anxiety, and depression, whereas men may exhibit more cough and sputum production. Imaging studies suggest that airway-predominant disease is more common in women, while men are more likely to demonstrate emphysema-predominant patterns. Furthermore, women face an increased risk of exacerbation, yet they are more likely to experience underdiagnosis or misdiagnosis. Treatment responses and comorbidity patterns also show sex- and gender-related variations. Despite these differences, most clinical guidelines and therapeutic strategies do not differentiate by sex and gender, highlighting a gap in personalized COPD management. Overall, growing evidence underscores the importance of incorporating sex and gender as biological and sociocultural variables in COPD research, diagnosis, and treatment. Recognizing sex/gender-specific risk profiles, symptom patterns, and disease phenotypes may improve early detection and enable more targeted, effective interventions. This narrative synthesis, derived from a meticulous search in PubMed and the critical selection of 74 articles from the 448 identified originally, integrates evidence from guideline statements, registry studies, mechanistic and preclinical research, imaging and physiology investigations, systematic reviews, and randomized controlled trials that report sex- and gender-disaggregated data.

## 1. Introduction

**Figure 1:**
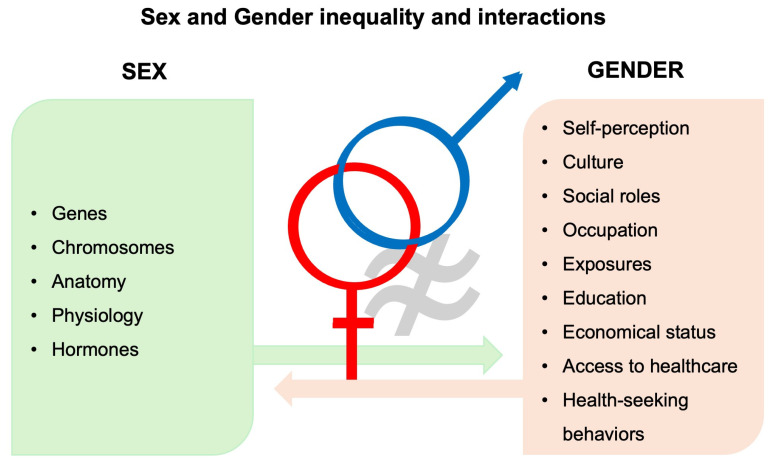
Sex and gender inequality and interaction.

Sex and gender are distinct but interrelated determinants of health. Sex, which should be considered a spectrum although research data are presented in binary terms, refers to biological attributes (chromosomes, genes, hormones, and anatomy), whereas gender refers to social and cultural constructs influencing behavior, exposures, and healthcare access [1,2,3,4] (Figure 1). Men and women are not identical in terms of respiratory disease and relevant healthcare. Sex influences airway geometry and lung structure, mucociliary and epithelial function, immunity and inflammation, metabolic responses, and susceptibility to infection, while gender shapes exposure profiles (e.g., cooking with biomass, cleaning occupations) and health-seeking behavior; both domains interact to determine phenotype, severity, and outcomes and affect the development, diagnosis, and management of respiratory diseases [5,6]. Thus, integrating sex/gender-aware perspectives is crucial for accurate phenotyping, equitable case detection, and personalized management.

Chronic obstructive pulmonary disease (COPD) remains a leading global cause of morbidity, ranking as the third leading global cause of death, affecting over 390 million individuals worldwide and accounting for more than 3 million deaths annually [7]. COPD has historically been considered a disease predominantly of older male smokers, reflecting past smoking patterns and occupational exposures [8,9]; however, over the last two decades, those epidemiological patterns have shifted. Since the early 2000s, the prevalence of COPD among women has risen steadily. Increased smoking rates, along with non-tobacco inhalational exposures—such as household air pollution from biomass fuels and certain occupational agents that disproportionately affect women—now contribute substantially to the COPD burden in low- and middle-income countries [10,11,12,13,14]. Women often develop COPD at younger ages and with fewer pack-years [15], experiencing greater dyspnea, anxiety, and functional limitation than men for the same FEV_1_ [16].

Despite mounting recognition of these differences, past research and many clinical trials have routinely not prespecified sex- and gender-stratified analyses, contributing to persistent gaps in evidence for sex- and gender-specific diagnostics and therapeutics [17]. To improve clinical care and advance precision respiratory medicine, contemporary research must collect information regarding reproductive history (e.g., pregnancy, menopausal status) and hormonal exposure, such as the use of hormonal contraception or hormone replacement therapy, ensure adequate female representation, and analyze outcomes by sex and gender as biological variables [17,18].

This review aims to synthesize classical knowledge and the most recent evidence on sex and gender differences in COPD, emphasizing: (1) epidemiological and exposure patterns; (2) structural, immunologic, and molecular mechanisms; (3) differences in clinical presentation, comorbidity, and prognosis; and (4) implications for diagnosis, treatment, and future research priorities. A literature search was conducted in PubMed up to November 2025 using the search terms sex and gender in combination with COPD. Eligible publications included case reports, case series, original research articles, review articles, letters to the editor, randomized controlled trials (RCTs), non-randomized trials, and prospective or retrospective cohort studies. The search was limited to articles published in English. The initial search identified 448 records. After removal of duplicates and screening of titles, abstracts, and full texts, 74 studies were included in the final manuscript. By focusing on sex- and gender-based contrasts in patients with COPD, we seek to increase awareness and provide practical guidance for clinicians and researchers to advance sex- and gender-informed care.

## 2. Epidemiology and Risk Factors

### 2.1. Global Trends and Changing Demographics

The prevalence of COPD among women has risen substantially in recent decades, varying widely by region. In high-income countries, female prevalence now equals or exceeds male prevalence, largely driven by tobacco use and biomass fuel exposure [19,20,21]. In North America, the prevalence of COPD in women equals prevalence in men (F: 7.30% vs. M: 8.07%) [22] and is rapidly increasing, especially in young women. In the United States, female COPD mortality surpassed that of men in 2021, making COPD the third leading cause of death among women [23]. Similar trends have been reported in Europe and Oceania [24], while the highest male prevalence has been observed in South East Asia (M: 11.34% vs. F: 5.2%). Regional differences may reflect actual biological and socioeconomic geographic variations, but they may also be influenced by diagnostic practices and data sources. In Europe, gender differences reflect smoking habits and variable occupational exposures, while in Asia and Africa, excessive biomass exposure and limited access to healthcare contribute to disease burden and severity [25]. Buttery et al. (2021) emphasized the disproportionate burden of COPD among women, who experience greater lung function loss, faster disease progression, higher symptom burden, more multimorbidity, and greater healthcare utilization despite historically lower cumulative tobacco exposure [26].

### 2.2. Tobacco Exposure and Susceptibility

Although tobacco smoking remains the principal risk factor for COPD, women appear more vulnerable to its deleterious effects. Data from the UK Biobank analyzing the association of airflow obstruction with cigarette smoking history in 149,075 women and 100,252 men showed this sex difference in tobacco susceptibility. Women who have ever smoked are at a greater risk of airflow obstruction than men who have ever smoked for equal exposure (duration, cigarettes per day, and pack-years), and the dose–effect relationship between airflow obstruction and smoking exposure is not linear for either sex. Women, both ex and current smokers, are at risk of airflow obstruction at lower doses of smoking, and even the risk reduction observed after quitting tend to be less evident in women for the same amount of time, although this difference was not statistically significant [27]. In a case–control study of 954 COPD patients, Sørheim et al. (2010) found that among two subgroups, those with early onset disease (<60 years old, *n* = 316) or low cumulative smoking exposure (<20 pack-years, *n* = 241), women had significantly lower predicted FEV_1_% (50.6% vs. 56.0%, *p* = 0.006 and 48.7% vs. 55.8%, *p* = 0.001, respectively) and more severe airflow limitation (41.7% vs. 31.1% in GOLD stage 3–4, *p* = 0.050 and 50.4% vs. 35.6% in GOLD stages 3–4, *p* = 0.02, respectively) compared with men [19]. These findings in these special populations may suggest an increased female susceptibility to smoke-induced airway damage. Similarly, of 2500 study subjects in the COPDGene study, a striking female predominance (66%, *p* < 0.0004) was shown in severe early onset disease defined as age < 55 yr and postbronchodilator FEV_1_% predicted less than 50% [20], while in the multivariable logistic regression analysis for the prediction of early onset COPD, the female sex retained a significant OR of 3.1 (95% CI, 1.1–8.7; *p* = 0.03). Interestingly, these younger patients had significantly less tobacco exposure compared with older COPD patients, supporting the hypothesis that gene-by-sex interactions, among other factors, may be more important in this subset of patients than the amount of smoking [20].

### 2.3. Non-Tobacco Exposures

In low- and middle-income countries, biomass smoke exposure from cooking and heating is a major COPD risk factor among women [13,28]. Prolonged indoor exposure to biomass fuels contributes to airway inflammation and remodeling, even in never-smokers [29]. A study in the Moroccan population showed a strong association between biomass exposure and the occurrence of COPD, independent of smoking; wood heating in men (OR = 3.8; 95% CI: 1.4 to 10.4) and biomass cooking in women (OR = 7.2; 95% CI: 1.7 to 30.0) [13]. Gendered occupational roles—such as textile work, agriculture, or healthcare—may also influence exposure to dusts, cleaning agents, and chemicals and are often under-recognized [30]. Regular use of chemical disinfectants and aromatic solvents predominates among women, while men are more commonly exposed to insecticides and organic and inorganic dusts, as they are more likely to work in agriculture, construction, or mining, often without protection and on top of smoking [31].

### 2.4. Genetic, Developmental, and Hormonal Factors

Sex-specific genetic susceptibility to COPD has been increasingly recognized. Emerging evidence implicates loci on the X chromosome and hormone-responsive genes involved in lung development and immune modulation as potential contributors to sex-related differences in disease risk [9,32]. In the COPDGene study, Foreman et al. (2011) reported that early life exposures, including maternal smoking (70% in early onset vs. 44% in older onset cases; *p* = 0.0001), maternal COPD (23% vs. 12%; *p* = 0.03), and low birth weight, may predispose females to a reduced airway caliber and an increased risk of obstructive disease in adulthood [20]. Notably, maternal COPD remained an independent predictor of early onset disease after adjustment (OR 4.7; 95% CI 1.3–17; *p* = 0.02), underscoring the potential importance of maternal and sex-linked genetic interactions in COPD pathogenesis [20].

## 3. Biological Mechanisms Underpinning Sex Differences

### 3.1. Airway Anatomy and Respiratory Mechanics

Corrected for height, females have a lower total lung capacity, tidal volume, minute ventilation, peak inspiratory and expiratory flow, airway size, trachea diameter, and pharyngeal airway length [33]. Females possess smaller airway lumen relative to the lung volume compared with males, resulting in a greater airway resistance and an earlier dynamic airway closure during exertion [34]. Imaging and physiological studies corroborate these structural differences, showing a lower emphysema burden but a greater small-airway dysfunction in women [35]. Collectively, these anatomical characteristics probably contribute to heightened dyspnea and ventilatory limitation at lower absolute workloads [6].

Potential biological mechanisms include smaller airway size leading to higher inhaled smoke concentration per breath, sex-related hormonal or metabolic variations in smoke detoxification, and differential immune or inflammatory responses between sexes [36].

Alternative explanations, though less likely, include diagnostic bias (with women more frequently misclassified as having asthma), reporting bias in smoking exposure, and selection bias related to participant recruitment in observational studies [37,38].

### 3.2. Hormonal Modulation

Sex hormones influence lung development, respiratory structure, and function. Estrogens can enhance mucociliary clearance and antioxidant defenses but may also amplify inflammatory responses under oxidative stress [39]. Progesterone modulates ventilatory control and respiratory drive, while androgens impact respiratory muscle mass [32]. Testosterone positively affects respiratory muscle strength, and higher testosterone levels have been correlated with better lung function in men [40]. Airways are influenced by hormonal fluctuations during menstrual cycle, puberty, pregnancy, and menopause, as well as by exogenous hormonal therapies. The menopausal transition represents an understudied period regarding its impact on physiological lung function decline and aging. It has been suggested that the decline in estrogen at menopause is linked to an increased risk of FEV_1_ decline and an increased symptom burden [9]. Hormonal replacement treatment (HRT) in menopause has been associated with new onset asthma, whereas its role is unclear in COPD [39]. There is no evidence that HRT impacts COPD incidence; however, it has been linked to FEV_1_ improvements in elderly women, while potential harmful associations with COPD exacerbations have been reported [41]. Overall, the available data are scarce and inconsistent; thus, further research is needed.

The pivotal role of hormones in the course of COPD has been highlighted in a large prospective cohort study from the UK Biobank, which investigated female reproductive history in relation to COPD outcomes [42]. Factors associated with an increased risk of COPD-related hospitalization or death included a parity greater than three (also linked to a lower FEV_1_/FVC ratio), late menarche (age > 15 years), early menopause (age < 47 years), polycystic ovary syndrome or ovarian cysts, any use of hormone replacement therapy (HRT), hysterectomy, and bilateral oophorectomy—though the latter two were paradoxically associated with better lung function. Notably, oral contraceptive use was the only factor associated with both a lower risk of COPD-related hospitalization or death and a higher FEV_1_/FVC ratio [42].

### 3.3. Immunologic and Molecular Dimorphism

Females generally mount stronger innate and adaptive immune responses, associated with increased production of inflammatory cytokines (e.g., IL-6, TNF-α) [43,44]. It is postulated that these may contribute to more frequent exacerbations and autoimmune comorbidities.

Several studies on bronchoalveolar lavage (BAL) have demonstrated remarkable differences between sexes. Macrophage autophagy was found to be increased in females with COPD [45], while distinct T-cell profiles exist, with Th1 inflammation correlating with goblet cell density and BAL macrophages in females and Th2 inflammation correlating with IgG serum concentration in male smokers [44].

### 3.4. Epigenetic and Chromosomal Effects

DeMeo et al. demonstrated sex-specific transcriptomic profiles in COPD lung tissue, implicating X-linked immune genes and epigenetic regulation. Escape from X-chromosome inactivation in females results in a higher expression of immune-related genes [9]. Sex-specific methylation patterns and microRNA expression may modulate COPD susceptibility and progression [39].

**Figure 2:**
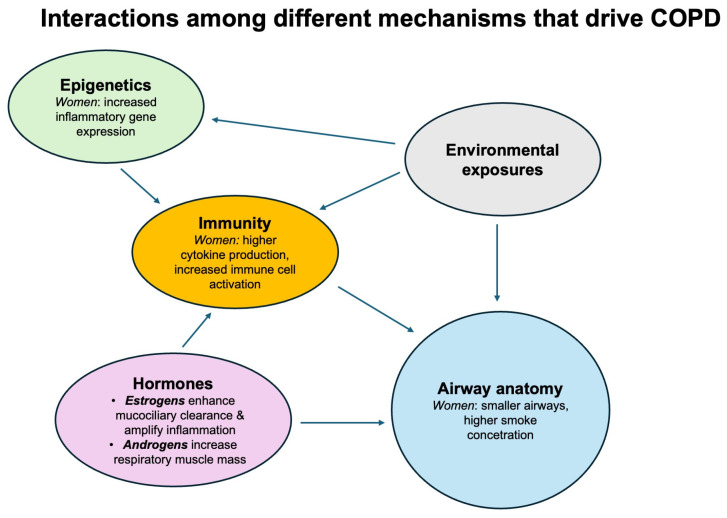
Integrated mechanisms driving chronic obstructive pulmonary disease (COPD).

In summary, anatomy, hormones, immunity, and epigenetics interact with each other and drive COPD. The respiratory system is vulnerable to environmental exposure, immunity, and chronic inflammation. Hormones modulate immune response and repair, while epigenetics alter gene expression and further maintain the self-perpetuating cycle of COPD (Figure 2).

## 4. Clinical Phenotypes and Functional Differences

### 4.1. Symptom Burden and Quality of Life

Women with COPD report greater dyspnea, fatigue, and psychological distress at comparable airflow limitation [26,46]. In the large TORCH study population, dyspnea and health-related quality of life scores were more impaired in women than men for a similar degree of physiologic impairment, especially in mild–moderate COPD. This heightened perception of dyspnea was reflected in a shorter time to first exacerbation and a higher number of exacerbations reported from women than men, but with a similar number of hospital admissions caused by exacerbations for both sexes [47]. Gut-Gobert et al. (2019) confirmed that women experience greater perceived breathlessness for a given ventilatory workload [36].

Physiological studies and large population-based studies show that differences in exertional dyspnea intensity between men and women may reflect biological differences in airway size, lung volumes, and respiratory muscle strength [48]. Women experience more shortness of breath and an increased frequency pattern of breathing as they tend to use a greater fraction of their ventilatory capacity to achieve the same absolute exercise intensity and ventilation compared with men [48]. Anxiety and depression, which are more prevalent in women, further exacerbate symptom perception and healthcare use [49,50]. Men, conversely, exhibit a higher prevalence of chronic cough and sputum production, consistent with more emphysematous phenotypes [51]. These differences underline the need for sex- and gender-specific symptom assessment tools.

### 4.2. Lung Function and Imaging Patterns

Longitudinal studies have revealed accelerated lung function decline among female smokers, even after adjusting for exposure [19,52]. Prescott et al. estimated that the excess loss of FEV_1_ per pack-year of smoking was between 7.4 and 10.5 mL in female smokers and between 6.0 and 8.4 mL in male smokers based on a large cohort with more than 13,000 subjects [53]. In the same direction, early onset COPD is more prevalent among women [20,54].

Accelerated decline in women is reflected in imaging, too. A prospective analysis from the ECLIPSE study showed that although women had less emphysema at baseline, a more rapid decline in lung density over time was observed [55]. CT-based analyses consistently show that men have greater emphysema volume, while women demonstrate thicker airway walls and greater air trapping [6,51]. Women with COPD have smaller airway lumen diameter due to airway thickening and less extensive emphysema compared to men [35,56]. These structural and functional disparities influence disease classification and progression. Specifically, complementary computed tomography (CT) analyses by Dransfield et al. (2007) revealed that, for comparable levels of airflow limitation, men exhibited more emphysema, whereas women exhibited greater airway-predominant disease instead of emphysema-dominant disease [51]. Additionally, men had more CT emphysema than women at all stages of COPD, and sex was an independent predictor of the percentage of low attenuation areas.

An increased risk of small-airway disease in women after chronic smoke exposure was also shown in a mouse model, where chronic smoke exposure in female mice was associated with increased small airway remodeling, distal airway resistance, and significant airway wall thickening compared to male mice; these effects were mediated by down-regulation of antioxidant genes, increased oxidative stress, and activation of TGF-β1. Pivotal hormonal influence was demonstrated by attenuation of these effects when ovariectomies were performed or after treatment with tamoxifen [57]. Together, these data support the existence of sex-dependent structural and pathological phenotypes in COPD.

### 4.3. Comorbidities

Women with COPD are more likely to have fractures, osteoporosis, depression, anxiety, and rheumatoid arthritis [26,58], whereas men more often have diabetes, kidney diseases, cardiovascular disease, and metabolic syndrome [58,59]. The pathophysiological underpinnings may involve sex hormone effects on bone metabolism and vascular function [6].

### 4.4. Exacerbations, Hospitalizations, and Mortality

Women tend to experience more frequent COPD exacerbations and healthcare utilization than men [58,60]. Kilic et al. reported that female COPD patients may be more prone to severe exacerbations, a higher number of hospitalizations, and longer lengths of stay. Among patients hospitalized due to exacerbations, women also exhibited more severe exacerbation events with respiratory failure compared with men [61]. In the ARCTIC real-world study (*n* > 17,000), Lisspers et al. reported that women had a higher number of moderate exacerbations compared with men (6.66 vs. 4.66, *p* < 0.01) and a 12% increased risk of experiencing an earlier exacerbation (HR = 1.12; 95% CI 1.09–1.16, *p* < 0.0001), while no significant difference was observed in severe exacerbations (2.59 vs. 2.56, *p* = 0.60) [58]. When focusing solely on COPD-related events, women had slightly more hospitalizations (0.17 vs. 0.14, *p* = 0.07), spent more nights in hospital (1.21 vs. 0.85, *p* < 0.001), and had more outpatient hospital visits (0.15 vs. 0.12, *p* = 0.15) [58]. In contrast, mortality was significantly higher in men (45% vs. 38%, *p* < 0.0001), with a shorter median survival time from COPD diagnosis to death (9.0 years, 95% CI: 8.75–9.30) compared with women (11.1 years, 95% CI: 10.8–11.5) [58].

Similarly, in a large cohort of 22,429 newly diagnosed COPD patients from UK primary care settings in a 10-year study period (2006–2016), 48% were women. Women, overall, were at a 17% higher risk for a first moderate or severe exacerbation, with a median time of 504 days vs. 637 for men. Interestingly, the risk was doubled in the younger 40 to 65 years old subgroup up to 35%, whereas it fell to 8% in ages over 65, reflecting age-related differences between the sexes [62]. More recently, Whittaker et al. (2025) analyzed over 28,000 hospital admissions for COPD in England and found that women were more likely to receive noninvasive ventilation and specialist review upon admission, while experiencing slightly lower 90-day mortality (OR 0.88, 95% CI 0.81–0.96, *p* = 0.03) [60]. These findings suggest that, despite higher morbidity, women may experience improved survival—potentially due to earlier healthcare contact and less extensive emphysematous destruction.

### 4.5. Diagnostic and Gender Bias

Gender bias remains a persistent challenge in women’s health research and clinical care, and more specifically in COPD [9,63]. In a community study, Moffett et al. (2025) found that women with spirometrically confirmed COPD were less likely than men to receive a clinical diagnosis (adjusted HR 0.66; 95% CI 0.50–0.88) [64]. In the same study, the mean time to diagnosis after spirometry was greater in women (106.5 months; 95% CI 86.2–127.7) than men (89.7 months; 95% CI 72.9–106.6) [64]. The mislabeling of chronic cough or dyspnea as asthma or anxiety is common in women, leading to delays in treatment.

Soriano and Polverino emphasized the importance of distinguishing sex (biological) from gender (sociocultural) when assessing respiratory symptoms [65]. Spirometry reference values should consider sex, height, and ethnicity, and clinicians should remain aware of implicit diagnostic bias.

## 5. Therapeutic Responses and Management Considerations

### 5.1. Pharmacologic Treatment

Most pharmacological trials have underrepresented women. Nevertheless, several studies have reported sex-specific differences in treatment outcomes. In the pooled IGNITE trials, Tsiligianni et al. demonstrated that indacaterol/glycopyrronium significantly improved lung function, dyspnea, rescue medication use, and symptoms in both sexes, with slightly larger improvements in health status observed among women [66]. Data from the Lung Health Study (*n* = 5887; 37% female) showed a larger, persistent bronchodilator response to ipratropium in females (6.0% vs. 2.9%), and the effect was more pronounced in subjects with a lower body mass index [67]. These sex-related changes in FEV_1_ were attributed to the greater gene expression for the M3 muscarinic receptor relative to M2 receptors in female compared to male lungs.

A systematic review by Rogliani et al., which included 23 randomized controlled trials and observational studies, reported that 28% of the available evidence indicated a better therapeutic response in men compared with women, although overall findings remained inconsistent [16]. Biological differences in the airway size, muscle mass, and pharmacokinetics may contribute to treatment response heterogeneity.

### 5.2. Inhaler Technique and Adherence

Calzetta et al. observed that women make more critical inhaler errors (OR 1.80; 95% CI 1.22–2.67), partly due to lower hand strength and device preference [68]. Education and device selection should thus consider ergonomic and behavioral factors.

Adherence may also differ by gender, with women more influenced by psychosocial barriers and body-image concerns regarding steroid use [65].

### 5.3. Pulmonary Rehabilitation and Lifestyle Interventions

Pulmonary rehabilitation benefits both sexes, though women often report greater improvement in dyspnea and emotional well-being [6,69]. However, caregiving responsibilities and travel constraints may reduce female participation, while tailored community- or home-based programs could improve access.

Successful smoking cessation has proven to be more difficult in women than in males who had higher sustained quit rates at 36 months, after adjustment for educational level and marital status. This is attributed to greater withdrawal symptoms and greater weight gain in women, as well as to more anxiety and nicotine dependence. Thus, nicotine replacement therapy has shown to be less effective in women while antidepressant medications for smoking cessation perform better, facts that highlight the need for potential sex and gender guidance for smoking cessation [70].

### 5.4. Precision and Sex-Specific Medicine

DeMeo proposed a sex-aware drug response [9]. Incorporating hormonal status, menopause, and testosterone deficiency into clinical phenotyping may refine the “omics” framework, integrating genomics, metabolomics, and proteomics to identify the biomarkers predictive of disease progression and personalized management strategies [9].

### 5.5. Health Services and Gendered Determinants

Gender roles and societal expectations significantly influence health behaviors and access to healthcare. Women, often primary caregivers, may delay seeking care, while men under-report symptoms due to stigma [69,71]. Economic inequities and differences in health literacy exacerbate disparities [72] and support the gender health paradox: women live longer but consistently report poorer physical and mental health than men [73]. Sodhi et al. highlighted the importance of integrating sex- and gender-based medicine in pulmonary training and clinical guidelines to mitigate these biases [18].

**Figure 3:**
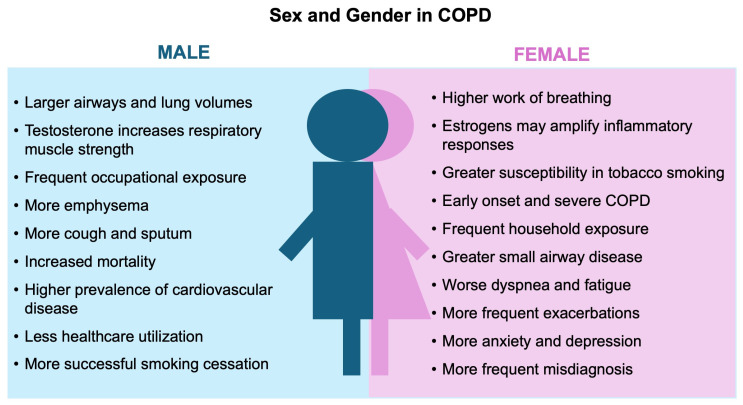
Sex- and gender-based differences in COPD.

Sex- and gender-based differences in COPD are pictured in Figure 3 and summarized in Table 1.

## 6. Research Perspectives and Future Directions

To advance equitable COPD care, research must systematically integrate sex and gender as disease determinants of major importance that may reveal different treatable traits. Key priorities proposed in order include:

i. Sex-stratified analyses in all clinical trials and registries to identify possible different risk factors, disease course and severity, and treatment outcomes.
ii. Inclusion of hormonal factors (e.g., menopause, hormone therapy, testosterone deficiency) in COPD phenotyping. The incorporation of the hormonal background of the patient in big registries may lead to alterations in the conventional approach of “one-size-fits-all”.
iii. Development of sex-specific reference standards for imaging and lung function to avoid misclassification.
iv. Design of interventions targeting gender-related barriers in correct and timely diagnoses, treatment adherence, and rehabilitation, with the ultimate goal of equal healthcare access combined with the appropriate personalized approach.
v. Intersectional analyses examining how sex and gender interact with age, ethnicity, and socioeconomic status to identify different risks and outcomes.
vi. Integration of multi-omics approaches to identify sex-specific biomarkers of risk and therapeutic response.

Emerging frameworks such as the “treatable traits” approach should explicitly incorporate sex and gender as clinical modifiers to optimize effective disease management [39].

Key take-home points, research gaps, recommendations, and clinical implications are presented in Table 2.

## 7. Conclusions

Sex and gender differences in COPD are robust, both clinically and biologically meaningful, and dynamic across the life course. Biological sex (airway geometry, hormones, immunology, metabolism) interacts with gender-related exposures (biomass, occupational inhalants, smoking patterns, obesity prevalence) to produce divergent phenotypes, symptom burdens, and comorbidity clusters. In the near future, clinicians should be able to apply sex- and gender-oriented case findings and diagnostic strategies, screen for relevant comorbidities (mental health, bone health), and ideally adopt tailored personalized behavioral interventions. To achieve this goal, research must prioritize sex-disaggregated designs, multi-omic endotyping, and sex-sensitive implementation science to enable precision respiratory medicine for both women and men. Doing so will enhance diagnostic accuracy, optimize therapeutic choices, and reduce disparities in respiratory outcomes.

## Figures and Tables

**Table 1:** Sex- and gender-based differences in COPD: an overview.

Domain	Characteristics	Key References
Epidemiology and life-course patterns	Historically male-dominant; now equal or female-dominant in many regions. Female prevalence and mortality are rising globally due to smoking and biomass exposure. Early onset and severe COPD is relatively more frequent in women, even with lower cumulative smoke exposure.	Soriano 2023 [65]; Whittaker 2025 [60]
Pathophysiology and mechanisms	Women show greater small-airway disease, less emphysema, higher airway reactivity, and faster FEV_1_ decline per unit exposure. Estrogen/testosterone balance influences oxidative stress and mitochondrial signaling. X-linked and hormonal mechanisms may enhance susceptibility.	DeMeo 2021 [32]; Milne 2024 [6]; Sørheim 2010 [19]
Hormonal and metabolic influences	Hormonal influences are less studied; post-menopausal hormonal decline may worsen lung aging. Emerging data suggest female sex hormones modify oxidative pathways and inflammation, though evidence remains limited.	Lenoir 2020 [40]; Reddy 2023 [39]
Clinical presentation and symptom burden	Women present with worse dyspnea and fatigue at comparable obstruction, more anxiety/depression, and higher exacerbation frequency. Men display greater radiologic emphysema and lower BMI. Symptom–severity discordance (more symptoms, less emphysema) is typical in women.	Lisspers 2019 [58]; Milne 2024 [6]
Diagnosis and bias	Women are less likely to receive COPD diagnosis, even with obstructive spirometry (documented bias). Misclassification as asthma or “bronchitis” is common, leading to delayed treatment.	Heise 2019 [71]; Moffett 2025 [64]; Soriano 2023 [65]
Gender and sociocultural determinants	Gender norms influence smoking patterns, fuel exposure, diagnosis, and access to care. Gender inequality correlates with underdiagnosis and delayed therapy in women globally.	Heise 2019 [71]; Lisspers 2019 [58]; Martinez 2012 [69]; Soriano 2023 [65]
Lung function and imaging phenotypes	For a given FEV_1_ impairment, men show more emphysema on CT; women show more airway-predominant disease. Women’s FEV_1_ declines per pack-year faster, supporting higher smoke susceptibility.	Dransfield 2007 [51]; Foreman 2011 [20]; Gan et al. 2006 [52]
Biomarkers and omics	Omic analyses reveal sex-specific transcriptomic, proteomic, and mitochondrial patterns; female COPD is associated with heightened oxidative stress and distinct inflammatory signatures.	DeMeo 2021 [32]; DeMeo 2025 [9]; Reddy 2023 [39]
Treatment response and pharmacology	Dual bronchodilators and inhaled therapies are generally effective across sexes, but women may perceive greater symptom relief. Women show distinct patterns of inhaler errors and adherence barriers. Need for sex-stratified dosing data.	Calzetta 2022 [68]; Tsiligianni 2017 [66]
Health-Care utilization, hospitalization, and outcomes	Women hospitalized for COPD exacerbations are more symptomatic but have lower short-term mortality and better readmission outcomes despite higher use of NIV and resources.	Dransfield 2007 [51]; Kilic et al. 2015 [61]; Lisspers 2019 [58]; Whittaker 2025 [60]
Therapeutic and precision-medicine implications	Incorporate sex as a biological variable in COPD trials; develop sex-specific endpoints and preventative strategies (smoke, biomass, occupational exposures).	DeMeo 2025 [9]; Milne 2024 [6]; Rogliani 2022 [16]

**Table 2:** Key take-home points, research gaps, recommendations, and clinical implications.

**Key take-home points** Women are increasingly affected by COPD, often at younger ages and lower exposures. Current smoking females, particularly beyond the perimenopausal period, experience a faster and more pronounced decline in lung function than men. Females experience greater dyspnea, anxiety, and osteoporosis; males show more emphysema and cardiovascular disease. Sex- and gender-aware diagnoses, management, and research design are essential for equitable precision-medicine approaches. **Research gaps and recommendations** Improve exposure assessment (biomass, occupational, ambient pollution) with gender-sensitive measures. Validate diagnostic algorithms and complementary tests (oscillometry, DLCO, CT quantitation) for airway-predominant disease common in women. Mandate sex-disaggregated reporting and prespecified sex-stratified analyses in COPD trials and cohorts. Leverage multi-omics (genomics, metabolomics, proteomics) with sex as a primary analytic variable to identify actionable endotypes. **Clinical implications** Clinicians should maintain a high index of suspicion for COPD in women, even in never-smokers, and should perform comprehensive assessments of exposure histories. Diagnostic tools should incorporate methods able to identify airway-predominant disease. A holistic approach of the patient should be adopted, with particular attention to the different incidence of comorbidities between men and women. Integration of sex- and gender-related differences into clinical practice represents a major step towards precision medicine in COPD.

## Data Availability

Extracted data are available from the corresponding author on request.

## Abbreviations

The following abbreviations are used in this manuscript:

BAL	Bronchoalveolar Lavage
CI	Confidence Interval
COPD	Chronic Obstructive Pulmonary Disease
CT	Computed Tomography
FEV_1_	Forced Expiratory Volume in 1 s
FVC	Forced Vital Capacity
HR	Hazard Ratio
HRT	Hormone Replacement Therapy
OR	Odds Ratio
TLC	Total Lung Capacity
